# Daily Oral Emtricitabine/Tenofovir Preexposure Prophylaxis and Herpes Simplex Virus Type 2 among Men Who Have Sex with Men

**DOI:** 10.1371/journal.pone.0091513

**Published:** 2014-03-17

**Authors:** Julia L. Marcus, David V. Glidden, Vanessa McMahan, Javier R. Lama, Kenneth H. Mayer, Albert Y. Liu, Orlando Montoya-Herrera, Martin Casapia, Brenda Hoagland, Robert M. Grant

**Affiliations:** 1 Gladstone Institute of Virology and Immunology, San Francisco, California, United States or America; 2 University of California, Berkeley, California, United States of America; 3 University of California San Francisco, San Francisco, California, United States of America; 4 Asociación Civil Impacta Salud y Educación, Lima, Peru; 5 Fenway Institute, Fenway Health, Boston, Massachusetts, United States of America; 6 Beth Israel Deaconess Medical Center, Boston, Massachusetts, United States of America; 7 Bridge HIV, San Francisco Department of Public Health, San Francisco, California, United States of America; 8 Fundación Ecuatoriana Equidad, Guayaquil, Guayas, Ecuador; 9 Asociación Civil Selva Amazónica, Iquitos, Peru; 10 Instituto de Pesquisa Clínica Evandro Chagas, Fundação Oswaldo Cruz, Rio de Janeiro, Brazil; University of Ottawa, Canada

## Abstract

**Background:**

In addition to protecting against HIV acquisition, antiretroviral preexposure prophylaxis (PrEP) using topical 1% tenofovir gel reduced *Herpes simplex* virus type 2 (HSV-2) acquisition by 51% among women in the CAPRISA 004 study. We examined the effect of daily oral emtricitabine/tenofovir (FTC/TDF) PrEP on HSV-2 seroincidence and ulcer occurrence among men who have sex with men (MSM) in the iPrEx trial.

**Methods:**

HSV-2 serum testing was performed at screening and every six months. Among HSV-2-seronegative individuals, we used Cox regression models to estimate hazard ratios (HRs) of HSV-2 seroincidence associated with randomization to FTC/TDF. We used multiple imputation and Cox regression to estimate HRs for HSV-2 seroincidence accounting for drug exposure. We assessed ulcer occurrence among participants with prevalent or incident HSV-2 infection.

**Results:**

Of the 2,499 participants, 1383 (55.3%) tested HSV-2-seronegative at baseline, 892 (35.7%) tested positive, 223 (8.9%) had indeterminate tests, and one test was not done. Of the 1,347 HSV-2-seronegative participants with follow-up, 125 (9.3%) had incident HSV-2 infection (5.9 per 100 person-years). Compared with participants receiving placebo, there was no difference in HSV-2 seroincidence among participants receiving FTC/TDF (HR 1.1, 95% CI: 0.8–1.5; *P* = 0.64) or among participants receiving FTC/TDF with a concentration of tenofovir diphosphate >16 per million viable cells (HR 1.0, 95% CI: 0.3–3.5; *P* = 0.95). Among participants with HSV-2 infection, the proportion with ≥1 moderate or severe ulcer adverse event was twice as high in the placebo vs. active arm (5.9% vs. 2.9%, *P* = 0.02), but there were no differences in the proportions with ≥1 clinical examination during which perianal or groin ulcers were identified.

**Conclusions:**

Tenofovir in daily oral FTC/TDF PrEP may reduce the occurrence of ulcers in individuals with HSV-2 infection but does not protect against HSV-2 incidence among MSM.

## Introduction

*Herpes simplex* virus type 2 (HSV-2) is the primary cause of genital ulcer disease worldwide. In 2003, an estimated 536 million people aged 15–49 years were living with the infection, with seroprevalence varying widely across settings and populations.[1] Most infected individuals are unaware of their infections.[2] In symptomatic infections, the virus causes painful ulcerative lesions that can take two to four weeks to heal in primary outbreaks, and recurrences can be frequent. The prevalence of HSV-2 infection in the general population ranges from 10 to 60 percent, with higher prevalences in female sex workers, men who have sex with men (MSM), and certain regions of the world. [3] Among human immunodeficiency virus (HIV)-infected populations, the estimated seroprevalence of HSV-2 is 60–95 percent, [3] and individuals coinfected with HIV have increased susceptibility to HSV-2 shedding and clinical manifestations of HSV-2 disease. [4] Observational studies have found a two-to-three-fold higher risk of HIV acquisition among individuals with HSV-2 infection, [5], [6] although randomized trials of HSV-2 treatment have not reduced HIV acquisition or transmission [7], [8].

In addition to protecting against HIV acquisition, antiretroviral preexposure prophylaxis (PrEP) using tenofovir has been shown to have a protective effect on HSV-2 in women. Pericoital 1% vaginal tenofovir gel reduced the risk of HSV-2 acquisition by 51% in women participating in the CAPRISA 004 study. [9] In fact, the protection against HSV-2 in that study was higher than the effect of the gel on HIV acquisition, and a mathematical model suggested that its effect on HSV-2 acquisition may have played a role in its success in protecting against HIV. [10] Oral tenofovir-based PrEP also reduced HSV-2 acquisition by 28% among heterosexual men and women who were HIV-negative and HSV-2-seronegative in the Partners PrEP study. [11] The anti-herpetic effects of tenofovir have since been confirmed in vitro [12].

Protection against HSV-2 could enhance the public health impact of tenofovir when used as an antiretroviral agent for HIV prevention or treatment. Daily oral emtricitabine/tenofovir (FTC/TDF) PrEP was shown to reduce HIV acquisition in MSM in the iPrEx trial, [13] but it is unknown whether tenofovir protects against HSV-2 acquisition or disease expression among MSM. Using data from the randomized iPrEx study, our primary aim was to determine the effect of daily oral FTC/TDF on HSV-2 seroincidence; secondarily, we examined the effect of FTC/TDF on ulcer occurrence. To inform the development of HSV-2 prevention interventions among MSM, we also aimed to identify demographic and behavioral risk factors for HSV-2 seroincidence in the iPrEx cohort.

## Materials and Methods

### Ethics statement

The iPrEx study (ClinicalTrials.gov: NCT00458393) was approved by the Committee on Human Research at the University of California, San Francisco, as well as local institutional review boards (IRBs) at each study site: Comité Institucional de Bioética, Asociación Civil Impacta Salud y Educación, Lima, Peru; Universidad San Francisco de Quito, IRB #1, Quito, Ecuador; Fenway Community Health Institutional Review Board, Boston, MA; Comissão de Ética para Análise de Projetos de Pesquisa, CAPPesq Hospital das Clínicas da Faculdade de Medicina da USP, São Paulo, Brazil; Comitê de Ética em Pesquisa, Hospital Universitario Clementino Fraga Filho/Universidade Federal de Rio de Janeiro, Rio de Janeiro, Brazil; Comitê de Ética em Pesquisa do Instituto de Pesquisa Clínica Evandro Chagas, Rio de Janeiro, Brazil; National IRB: Comissão Nacional de Ética em Pesquisa – CONEP, Ministério da Saúde, Brasília, Brazil; University of Cape Town Research Ethics Committee, Cape Town, South Africa; Human Experimentation Committee, Research Institute for Health Sciences, Chiang Mai, Thailand; Ethical Review Committee for Research in Human Subjects, Department of Medical Services, Ministry of Public Health, Nonthaburi, Thailand; Research Ethics Committee, Faculty of Medicine, Chiang Mai University, Chiang Mai, Thailand. Written informed consent was obtained from each participant prior to enrollment in the study.

### Study population and procedures

Details of the iPrEx trial have been previously published. [13] Briefly, the study enrolled 2,499 MSM and transgender women at risk for HIV infection at 11 sites in Peru, Ecuador, Brazil, South Africa, Thailand, and the United States. Participants were randomized to receive either daily oral FTC/TDF or placebo. Monthly visits included medical history and symptom-directed physical examination, adverse event (AE) assessment, study drug dispensation, HIV testing, risk-reduction counseling, and adherence assessment. Serologic testing for HSV-2 and physical examinations for signs of sexually transmitted infections (STI) were performed by clinicians at screening (baseline), every six months during follow-up, and when the study drug was suspended, or when prompted by symptoms reported during the monthly medical examination. HIV infection status was determined using two rapid antibody tests and confirmed by Western blot or RNA testing. Sexual practices during the previous three months were assessed by interviewer-administered questionnaires at screening and quarterly visits during follow-up. The primary analysis of iPrEx data included visits through the pre-specified cutoff date of May 1, 2010, while the current analyses include follow-up visits through September 30, 2010, the last visit at which participants would have been expected to have had exposure to study drug.

HSV-2 infection status was determined using ELISA (HerpeSelect, Focus Diagnostics). A negative HSV-2 test was defined as having an index ratio (i.e., the ratio of the optical density of the color generated by the sample to the optical density of a standard calibrator; IR) <0.9, while a positive HSV-2 test was defined as having an IR ≥3.5. [14] Tests with an IR ≥0.9 and <3.5 were classified as indeterminate; participants with an IR <3.5 were retested at the next testing time point. The date of HSV-2 seroconversion was the date of the first positive HSV-2 test. Perianal and groin ulcers were recorded if there was any ulcerative lesion identified during STI examination, which may have included ulcers associated with herpes, syphilis, chancroid, lymphogranuloma venereum, or excoriation. Ulcer AEs were identified by a clinician as an increase in ulcer severity or frequency from baseline, including new onset of ulcers in individuals without preexisting ulcer disease; severity was defined according to the infection criteria in the National Institutes of Health Division of AIDS Table for Grading the Severity of Adult and Pediatric Adverse Events, December 2004. Only clinical AEs that were Grade 2 or above were reported in iPrEx, per protocol; thus, Grade 1 ulcers were not reported as ulcer AEs. Ulcer AEs were identified during STI examination, or were self-reported by participants and not confirmed on clinical examination if symptoms resolved before the examination visit. Additionally, ulcers that were self-reported but confirmed to be a different clinical manifestation during examination were not classified as ulcers or included in this analysis. In a subset of participants assigned to the active arm, levels of FTC and tenofovir were measured in plasma and peripheral blood mononuclear cells (PBMCs). Participants with drug level tests were 1) in the DEXA substudy, which evaluated the impact of FTC/TDF on bone and body composition at seven sites in Peru, Brazil, South Africa, Thailand, and the United States; and/or 2) matched active-arm controls in the case-control substudy of HIV seroconverters (from nine iPrEx sites with active-arm seroconversions). Approximately one-third of the cohort had at least one drug level test.

### Statistical analyses

For HSV-2 prevalence analyses, the dependent variable was HSV-2 status at the screening visit, and independent variables were randomization group and other baseline characteristics, including age, level of education, transgender identity, number of alcoholic drinks on days when drinking in the past month, and sexual behaviors in the past three months. Sexual behavior variables were number of anal sex partners, any receptive anal intercourse with a condom (cRAI), any receptive anal intercourse with no condom (ncRAI), any insertive anal intercourse with a condom (cIAI), and any insertive anal intercourse with no condom (ncIAI). We used chi-square tests and log-binomial models to identify factors associated with HSV-2 prevalence at baseline. We also examined the association of age with HSV-2 prevalence and HSV-2 seroincidence using chi-square tests for trend.

For HSV-2 seroincidence analyses, the dependent variable was time to HSV-2 seroconversion during follow-up, and independent variables were randomization group and the demographic and behavioral variables examined in HSV-2 prevalence analyses. We calculated crude rates of HSV-2 seroincidence by randomization group and baseline demographic and behavioral characteristics, including only participants who tested seronegative for HSV-2 at the screening visit. Person-time at risk included time from study enrollment to the first of HSV-2 infection, study drug discontinuation, or loss to follow-up. We estimated the time-to-event distribution by randomization group using Kaplan-Meier methodology. We used Cox regression models to estimate unadjusted and adjusted hazard ratios (HRs) for time to HSV-2 seroincidence. An intent-to-treat analysis included only randomization group; to identify factors associated with time to HSV-2 seroincidence, a multivariable model additionally included demographic and behavioral variables that were statistically significant at the *P*<0.05 level in unadjusted analyses. Sexual behavior variables were time-updated in models at approximately three-month intervals, while other covariates were treated as fixed. All models were stratified by study site.

Among the same participants, we also conducted an as-treated analysis that accounted for study drug use among participants receiving FTC/TDF. Because drug level testing was only conducted in a subset of participants and visits, drug levels were imputed for participants in both arms at any monthly visit missing drug level data using chained equations and predictive means matching. Predictors in the imputations included study week, study site, baseline number of sexual partners, baseline ncRAI, transgender identity, body mass index, weight, report of an STI in the six months before screening, secondary education, circumcision, baseline HSV-2 infection, age, and number of drinks on days when the participant drank in the prior month. Covariates were used to predict the probability of having detectable drug and the probability that the level of tenofovir diphosphate (TFV-DP) in PBMCs was >16 fmol per million viable cells, the concentration associated with an estimated 90% reduction in HIV acquisition. [15] Drug levels were multiply imputed [16] for visits at which drug level testing was not conducted but the participant was still taking study drug, with 200 imputations per observation. [17] We then used site-stratified Cox regression to estimate HRs for HSV-2 seroincidence associated with being randomized to the FTC/TDF arm and having detectable drug with TFV-DP ≤16 or being randomized to the FTC/TDF arm and having detectable drug with TFV-DP >16. Unadjusted models included only a time-dependent covariate for drug detection, while adjusted models also included age, level of education, transgender identity, number of alcoholic drinks on days when drinking in the past month, and sexual behaviors in the past three months (number of anal sex partners, cRAI, ncRAI, cIAI, and ncIAI). Sexual behavior variables were time-updated at approximately three-month intervals.

To examine the effect of FTC/TDF on HSV-2 disease expression, we analyzed the occurrence of ulcers among those who tested seropositive for HSV-2 at baseline or during follow-up. To eliminate the potential effect of HIV infection on ulcer occurrence, participants were excluded if the HSV-2 diagnosis occurred at or after HIV seroconversion, and ulcers were excluded if they occurred at or after HIV seroconversion. We estimated the proportion of participants with ≥1 ulcer AE classified as Grade 2 or above (i.e., moderate, severe, or potentially life-threatening), ≥1 STI examination during which a perianal ulcer was detected, and ≥1 STI examination during which a groin ulcer was detected, using chi-square tests to compare proportions by randomization group. We assessed the proportion of visits at which symptoms were reported that prompted an STI examination, using a chi-square test for comparison by randomization group. We also examined ulcers occurring after HIV seroconversion to determine whether there were differences in ulcer occurrence by randomization group in the absence of study drug.

All analyses were conducted in SAS 9.3 or Stata 12.

## Results

### Study participants

Characteristics of the 2,499 iPrEx participants have been described previously. [13] Briefly, all participants were born male and 313 (13.0%) identified as transgender or as women. The mean age at enrollment was 25 years (range 18–67), and the majority of participants were enrolled at the three study sites in Peru (1,400, 56.0%). At baseline, over half of participants (59.4%) reported having had ncRAI in the past three months. Among participants with prevalent or incident HSV-2 infection, 11.6% used acyclovir or valacyclovir during study follow-up.

### HSV-2 prevalence

**Figure 1:**
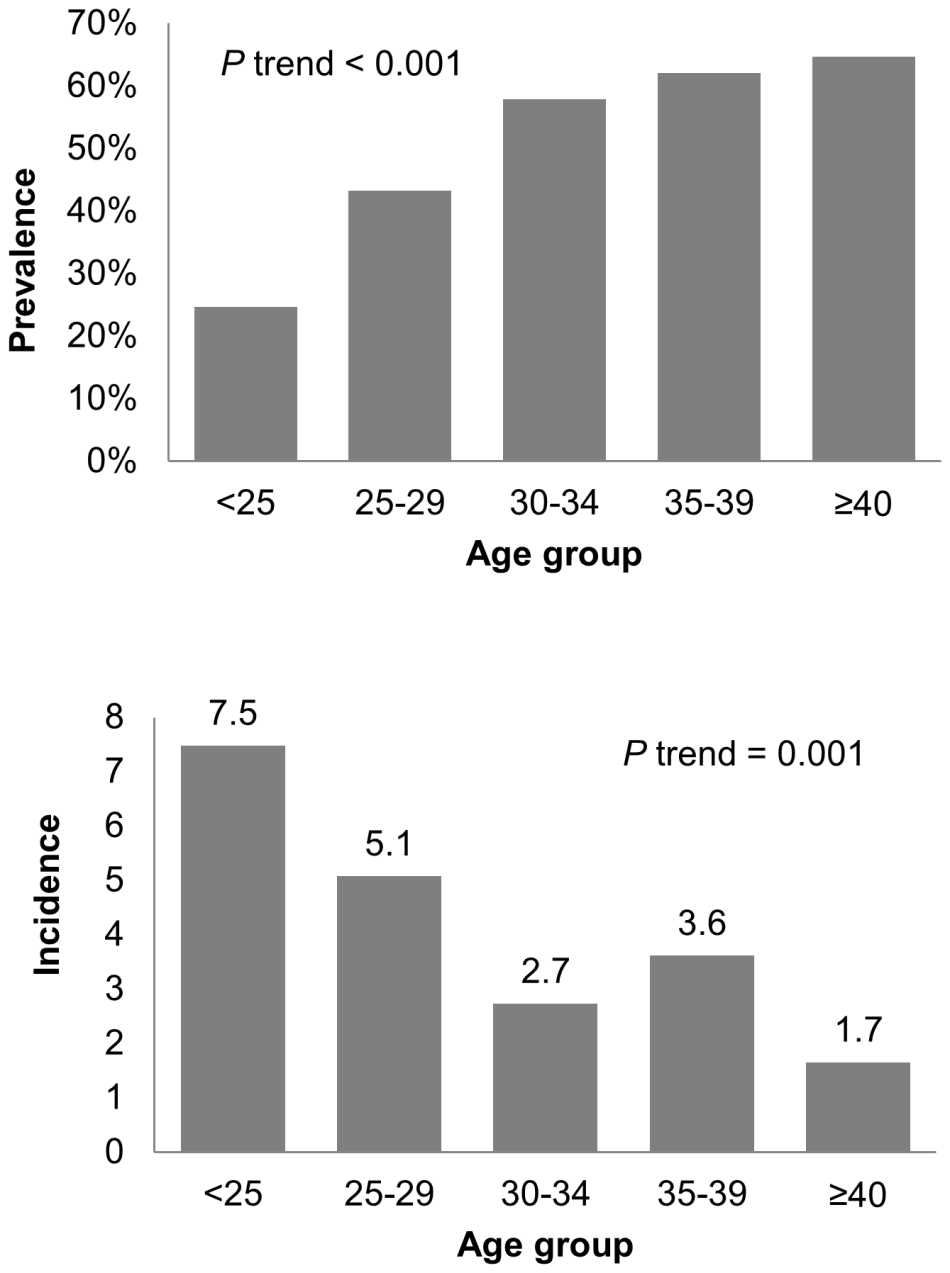
Baseline prevalence of HSV-2 and incidence of HSV-2 during study follow-up by age at enrollment. Figure 1a shows HSV-2 prevalence at baseline by age group at enrollment, while Figure 1b shows HSV-2 incidence during follow-up by age group at enrollment. HSV-2, herpes simplex virus type 2.

Of the 2,499 participants, 1383 (55.3%) tested negative for HSV-2 at baseline, 892 (35.7%) tested positive, 223 (8.9%) had indeterminate tests, and one test was not done. Of the 223 with indeterminate tests at baseline, 114 (51.1%) tested positive for HSV-2 infection at some point during follow-up. Factors associated with testing seropositive for HSV-2 at baseline included older age (*P* trend <0.001; Figure 1a), transgender identity (prevalence ratio [PR] 2.0, 95% confidence interval [CI]: 1.8–2.2; *P*<0.001), and not having a secondary education (PR 1.4, 95% CI: 1.2–1.5; *P*<0.001). The prevalence of HSV-2 infection was highest among participants living in Peru (46.0%), Brazil (37.8%), and Ecuador (37.3%), with lower prevalence among participants living in Thailand (6.4%), South Africa (17.6%), and the United States (27.1%; *P*<0.001). Randomization group was not associated with HSV-2 prevalence at baseline (*P* = 0.44). In multivariable analysis, all factors remained significantly associated with HSV-2 prevalence with the exception of level of education.

### FTC/TDF and time to HSV-2 seroincidence

Characteristics of 1,383 participants who tested seronegative for HSV-2 at baseline are presented by randomization group in Table 1. There were no differences in baseline characteristics by randomization group, with the exception of cRAI in the past three months being reported more frequently in the placebo arm (*P* = 0.01).

**Table 1:** Characteristics of participants testing HSV-2 seronegative at baseline by randomization group.^a^

	FTC/TDF (n = 692)	Placebo (n = 691)	P-value
Age group			0.11
<25	413 (60)	449 (65)	
25–29	139 (21)	123 (18)	
30–34	61 (9)	52 (8)	
35–39	28 (4)	34 (5)	
≥40	51 (7)	33 (5)	
Completed secondary education			0.38
Yes	557 (82)	571 (84)	
No	126 (18)	114 (17)	
Transgender identity			0.50
Yes	40 (6)	46 (7)	
No	652 (94)	645 (93)	
No. alcoholic drinks on drinking days, past month			0.11
0–4	323 (48)	294 (43)	
≥5	354 (52)	383 (57)	
Number of anal sex partners, past 3 months			0.87
0–1	90 (13)	85 (12)	
2–5	291 (42)	287 (42)	
≥6	311 (45)	319 (46)	
Insertive anal intercourse with condom, past 3 months			0.94
Yes	343 (50)	350 (51)	
No	349 (50)	341 (49)	
Insertive anal intercourse with no condom, past 3 months			0.57
Yes	419 (61)	408 (59)	
No	273 (39)	283 (41)	
Receptive anal intercourse with condom, past 3 months			0.01
Yes	292 (42)	340 (49)	
No	400 (58)	351 (51)	
Receptive anal intercourse with no condom, past 3 months			0.11
Yes	329 (48)	358 (52)	
No	363 (52)	333 (48)	

a HSV-2, herpes simplex virus type 2. Ns may not add up to column totals due to missing data.

Of the 1,383 participants who tested seronegative for HSV-2 at baseline, 36 (2.6%) did not contribute person-time to incidence analyses because they were retrospectively found to be HIV-infected at baseline, tested seropositive for HSV-2 at the enrollment visit subsequent to screening, or were lost to follow-up after enrollment. Of the remaining 1,347 seronegative participants, 125 (9.3%) were diagnosed with HSV-2 during follow-up, representing an incidence of 5.9 per 100 person-years (Table 2). In unadjusted analysis, HSV-2 incidence decreased with age, with the highest rate among participants aged <25 years (7.1 per 100 person-years) and the lowest rate among participants aged ≥40 years (1.6 per 100 person-years; *P* trend  = 0.001). Country of residence was also associated with HSV-2 incidence, with the highest rate among participants living in Ecuador (9.7 per 100 person-years) and the lowest rate among participants living in Thailand (1.7 per 100 person-years). The only behavioral factor associated with time to HSV-2 incidence was ncRAI in the past three months (HR 2.0, 95% CI: 1.4-3.0; *P*<0.001). In multivariable analysis, younger age and ncRAI remained associated with time to HSV-2 incidence, while level of education, transgender identity, alcohol use, and other sexual behaviors were not associated with time to HSV-2 seroincidence.

**Table 2:** Baseline characteristics by time to HSV-2 seroincidence.^a^

	n (%)	Events/PY	Incidence density	Unadjusted HR^b^ (95% CI)	Adjusted HR (95% CI)	P-value
	1347 (100)	125/2134	5.9			
Age group						0.02
<25	809 (60)	94/1322	7.1	1	1	
25–29	271 (20)	21/412	5.1	0.7 (0.4, 1.1)	0.7 (0.4, 1.1)	
30–34	121 (9)	5/188	2.7	0.3 (0.1, 0.8)	0.3 (0.1, 0.8)	
35–39	63 (5)	3/87	3.5	0.4 (0.1, 1.3)	0.4 (0.1, 1.5)	
≥40	83 (6)	2/125	1.6	0.2 (0.0, 0.7)	0.2 (0.0, 0.8)	
Completed secondary education						
Yes	1103 (83)	107/1741	6.1	1.3 (0.8, 2.2)		
No	230 (17)	18/362	5.0	1		
Transgender identity						
Yes	85 (6)	10/126	8.0	1.5 (0.8, 2.9)		
No	1262 (94)	115/2008	5.7	1		
Treatment assignment						
FTC/TDF	671 (50)	65/1062	6.1	1.1 (0.8, 1.5)	1.2 (0.8, 1.7)	0.41
Placebo	676 (50)	60/1071	5.6	1	1	
No. alcoholic drinks on drinking days, past month						
0–4	608 (46)	56/926	6.0	1		
≥5	711 (54)	65/1155	5.6	0.9 (0.6, 1.3)		
Number of anal sex partners, past 3 months^c^						
0–1	169 (13)	18/231	7.8	1		
2–5	563 (42)	42/846	5.0	0.6 (0.4, 1.1)		
≥6	615 (46)	65/1056	6.2	0.7 (0.4, 1.4)		
Insertive anal intercourse with condom, past 3 months						
Yes	681 (51)	52/1060	4.9	0.9 (0.9, 1.0)		
No	666 (49)	73/1074	6.8	1		
Insertive anal intercourse with no condom, past 3 months						
Yes	802 (60)	77/1304	5.9	1.0 (0.7, 1.4)		
No	545 (40)	48/829	5.8	1		
Receptive anal intercourse with condom, past 3 months						
Yes	617 (46)	67/961	7.0	1.0 (1.0, 1.0)		
No	730 (54)	58/1172	4.9	1		
Receptive anal intercourse with no condom, past 3 months						
Yes	671 (50)	86/1091	7.9	2.0 (1.4, 3.0)	2.0 (1.3, 2.9)	<0.001
No	676 (50)	39/1042	3.7	1	1	

a HSV-2, herpes simplex virus type 2; PY, person-years; HR, hazard ratio. Among participants testing HSV-2 seronegative at baseline. Ns may not add up to column totals due to missing data.

b Unadjusted HRs were derived from univariable models, while adjusted HRs were derived from multivariable models including variables that were statistically significant at the *P*<0.05 level in unadjusted analyses. Models were stratified by study site.

c Sexual behavior variables are shown at baseline for incidence estimates and were time-updated in models.

**Figure 2:**
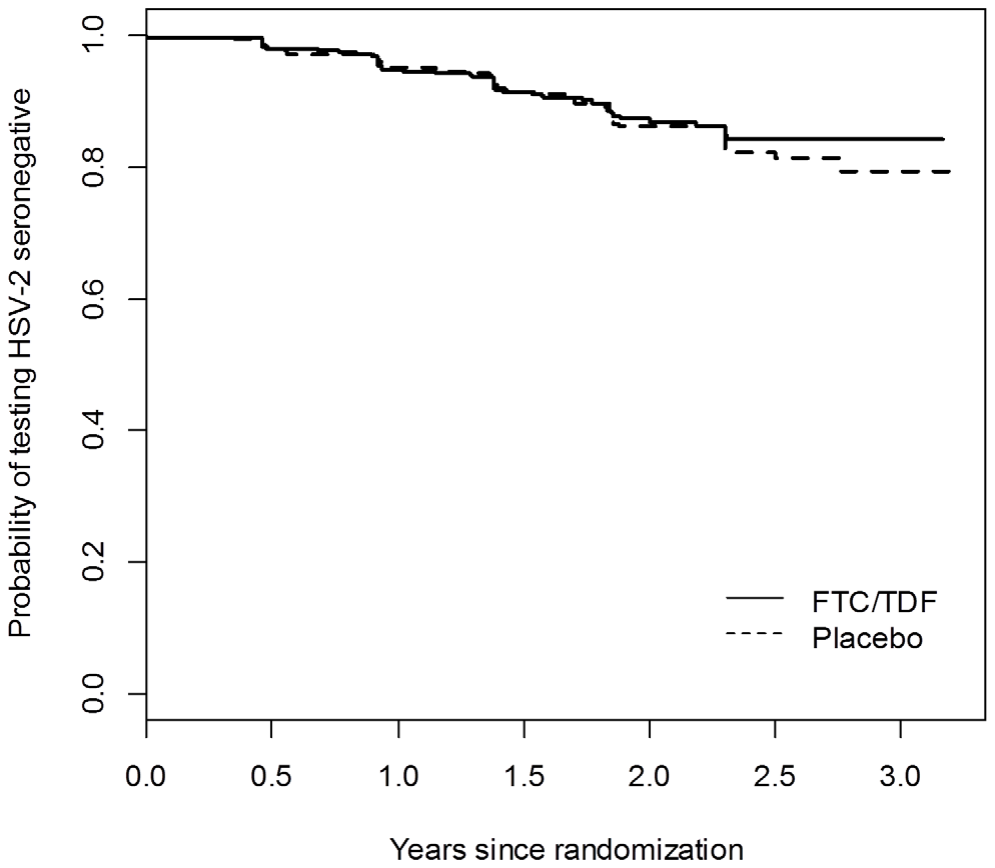
Probability of testing HSV-2 seronegative by randomization group. By Kaplan-Meier analysis. HSV-2, herpes simplex virus type 2.

Of those who acquired HSV-2 during follow-up, 65 were in the FTC/TDF group (incidence of 6.1 per 100 person-years) and 60 were in the placebo group (incidence of 5.6 per 100 person-years). There was no significant difference in time to HSV-2 incidence among participants assigned to the FTC/TDF arm compared with those assigned to the placebo arm (HR 1.1, 95% CI: 0.8–1.5; *P* = 0.64; Figure 2). Compared with participants in the placebo arm, there was also no difference in time to HSV-2 incidence among participants in the FTC/TDF group with a detectable drug level and ≤16 TFV-DP (HR 1.0, 95% CI: 0.4–2.5; *P* = 0.97), with similar results among those in the FTC/TDF group with >16 TFV-DP (HR 1.0, 95% CI: 0.3–3.5; *P* = 0.95). Results did not change after adjustment for age, education, transgender identity, alcohol use, or sexual behaviors.

### FTC/TDF and ulcer occurrence

**Figure 3:**
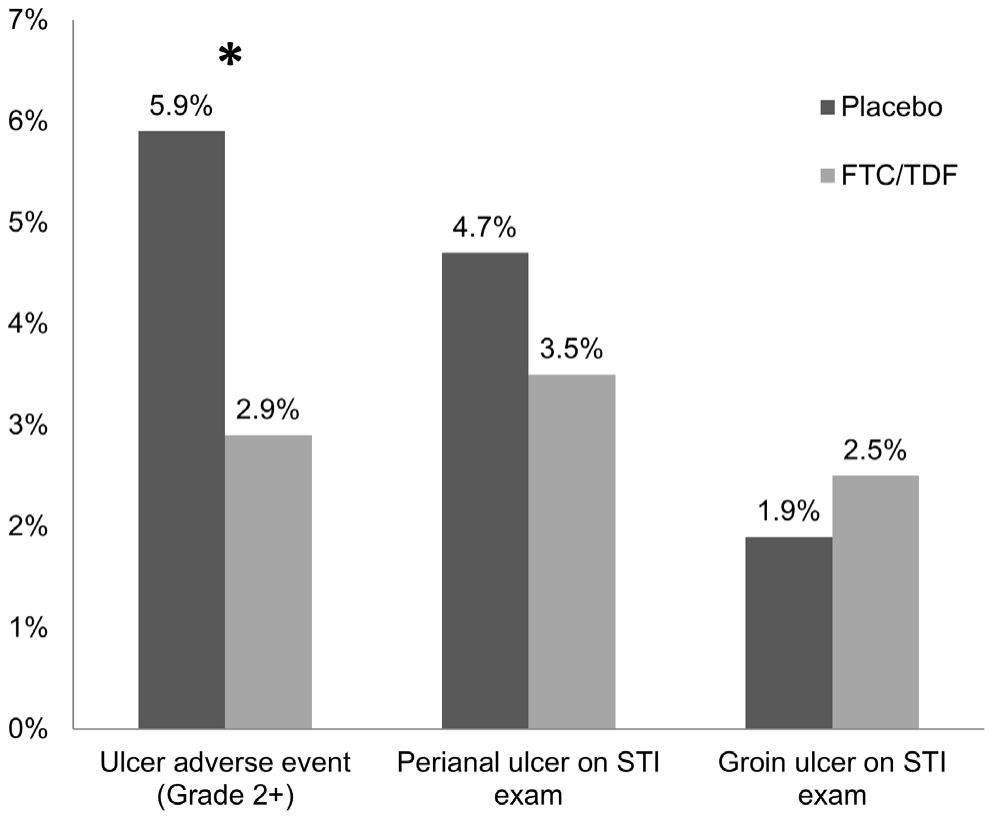
Proportion of prevalent or incident HSV-2 cases with ≥1 ulcer by randomization group. HSV-2, herpes simplex virus type 2; STI, sexually transmitted infection. Asterisk indicates *P*<0.05.

A total of 1,019 participants tested seropositive for HSV-2 at baseline or during follow-up; of those, 22 (2.2%) tested seropositive for HSV-2 after HIV seroconversion. Among the remaining 997, there were 72 ulcer AEs classified as Grade 2 or above, with 43 participants (4.3%) having ≥1 ulcer AE. Among the 72 ulcer AEs, 23 (31.9%) were confirmed on STI examination; for the remainder, symptoms resolved before an examination was conducted. Compared with participants in the placebo arm, the proportion of participants with ≥1 ulcer AE was reduced by half among participants in the FTC/TDF arm (2.9% vs. 5.9%, *P* = 0.02; Figure 3). There were no differences by randomization group in the proportion of participants with ≥1 STI examination during which a perianal ulcer (FTC/TDF 3.5% vs. placebo 4.7%, *P* = 0.37) or groin ulcer (FTC/TDF 2.5% vs. placebo 1.9%, *P* = 0.51) was identified; results were similar after excluding participants with a positive syphilis rapid plasma reagin test at the same visit. However, symptoms that prompted STI examination were less common in the FTC/TDF arm compared with the placebo arm (3.7% vs. 7.4%, *P* = 0.01).

Among the 89 participants with prevalent or incident HSV-2 infection who also seroconverted to HIV during study follow-up, 6.7% had ≥1 ulcer AE, 6.7% had ≥1 STI examination during which a perianal ulcer was identified, and 5.6% had ≥1 STI examination during which a groin ulcer was identified after HIV seroconversion, and thus after stopping study drug. The proportions with each type of ulcer did not differ between participants in the FTC/TDF arm and participants in the placebo arm.

Finally, the iPrEx protocol did not use the HSV-2 test manufacturer's suggested cutoffs for indeterminate (IR ≥0.9 and ≤1.1) or positive (IR>1.1) tests, but conducting the incidence and ulcer analyses using the cutoffs from the package insert yielded similar results.

## Discussion

In this analysis of participants in the iPrEx trial of daily oral FTC/TDF PrEP, we found no association between FTC/TDF and incidence of HSV-2 infection, even after accounting for actual use of FTC/TDF using drug level test results in a subset. The proportion of participants in the FTC/TDF arm with at least one ulcer AE classified as Grade 2 or above was half of that seen in the placebo arm, and this association was no longer present among participants who had stopped study drug after HIV seroconversion; however, this finding was not confirmed by ulcers identified during STI examinations and may have included ulcers of non-herpetic etiologies. In contrast to the 51% reduction in HSV-2 incidence among women randomized to use a 1% tenofovir topical gel in CAPRISA 004, [9] our results suggest that tenofovir in daily oral FTC/TDF may reduce the occurrence of ulcers in individuals with HSV-2 infection but does not protect against HSV-2 incidence among MSM.

The difference between the effects on HSV-2 incidence seen in CAPRISA and iPrEx may be due to differences in the route of transmission, method of drug delivery, or level of drug exposure. We found that the primary risk factor for incident HSV-2 infection in iPrEx was receptive anal intercourse without a condom, a finding that has been reported in several studies of behavioral risk factors for HSV-2 acquisition in MSM. [18], [19], [20] The rectal mucosa and cervicovaginal mucosa may differ in their susceptibility to HSV-2 infection. Additionally, although oral dosing of tenofovir achieves drug concentrations that are 20–100 times higher in rectal tissue than in vaginal and cervical tissue, [21], [22] topical application of tenofovir achieves a more than 100-fold higher concentration of the drug in the genital tract than oral dosing; [23], [24] furthermore, the inhibitory concentration for tenofovir is substantially higher for HSV-2 relative to HIV. [12] Drug concentration is also affected by adherence; while iPrEx participants reported taking over 90% of study drug doses, drug was detectable in the blood specimens of only 50% of participants tested in a random subsample.[13] Although we did not observe an effect of FTC/TDF even after accounting for drug levels, it may be that oral FTC/TDF will be shown to have an impact on HSV-2 incidence in settings where drug exposure is higher as a result of more consistent pill taking, such as the Partners PrEP study.[11]

We found that FTC/TDF was associated with a reduction in moderate or severe ulcer AEs among participants with HSV-2 infection, although this was not confirmed by clinical examination findings. Given the inhibition of HSV-2 replication observed after administration of tenofovir, it is biologically plausible that FTC/TDF reduced the frequency or severity of ulcers. Unlike acyclovir, tenofovir does not require the presence of the herpes virus for drug activation, suggesting that it may suppress ulcers before phosphorylation occurs. However, topical dosing may be required to achieve a concentration of drug in tissue sufficient to inhibit HSV-2 shedding, [12] and a study of adults coinfected with HIV and HSV-2 found no impact of oral tenofovir on rates of HSV-2 shedding. [25] More information is needed about the impact of oral and topical tenofovir on the clinical expression of HSV-2 infection.

There are several limitations of our analysis. First, although our intent-to-treat incidence analysis by treatment arm was strengthened by randomization, our findings may have been diluted by low levels of adherence among participants. While drug levels were not available for all participants or visits, we were able to conduct an incidence analysis that accounted for drug exposure but was subject to the limitations of multiple imputation of a substantial amount of missing data, potential unmeasured confounding, and wide confidence intervals. Of participants with prevalent or incident HSV-2, a small proportion were prescribed acyclovir or valacyclovir during study follow-up; if use of these medications biased our analysis toward the null, it is possible that the effect of FTC/TDF on ulcers is stronger than what we observed in our study. Our behavioral risk factor analysis included number of anal sex partners, position during anal sex, and condom use in the last three months, but we did not include oral sex as a potential risk factor for HSV-2 seroincidence; however, because HSV-2 is infrequently transmitted through oral sex, we expect this had a negligible effect on our analysis. There may have been some misclassification of HSV-2 results, particularly among participants with HSV-1 antibody, [26] but there is no expectation that this would differ by randomization arm. Some ulcer AEs were identified by self-report, which may be subject to inaccuracy or low sensitivity for HSV-2 recurrences, and we were not able to use HSV PCR to confirm that ulcers were herpetic. Finally, AEs only captured ulcers that increased in severity or frequency; thus, the reduction in ulcers associated with FTC/TDF may have been greater than what we observed.

To our knowledge, this is the first analysis of the effect of daily oral FTC/TDF PrEP on HSV-2 incidence and ulcer occurrence among MSM. In our study, oral FTC/TDF did not reduce the acquisition of HSV-2 infection. Although we found that FTC/TDF was associated with a reduction in ulcer AE occurrence, this finding was not confirmed by ulcers identified on clinical examination and should be replicated in settings where ulcer etiology can be confirmed.

## References

[1] Looker KJ, Garnett GP, Schmid GP (2008) An estimate of the global prevalence and incidence of herpes simplex virus type 2 infection. Bulletin of the World Health Organization 86: : 805–812, A 10.2471/BLT.07.046128 PMC2649511 18949218

[2] Xu F, Sternberg MR, Gottlieb SL, Berman SM, Markowitz LE, et al (2010) Seroprevalence of herpes simplex virus type 2 among persons aged 14–49 years — United States, 2005-2008. MMWR 59: 456–459. 20414188

[3] Gupta R, Warren T, Wald A (2007) Genital herpes. Lancet 370: 2127–2137. 18156035 10.1016/S0140-6736(07)61908-4

[4] Paz-Bailey G, Ramaswamy M, Hawkes SJ, Geretti AM (2007) Herpes simplex virus type 2: epidemiology and management options in developing countries. Sexually transmitted infections 83: 16–22. 17098770 10.1136/sti.2006.020966 PMC2598582

[5] Wald A, Link K (2002) Risk of human immunodeficiency virus infection in herpes simplex virus type 2-seropositive persons: a meta-analysis. The Journal of infectious diseases 185: 45–52. 11756980 10.1086/338231

[6] Freeman EE, Weiss HA, Glynn JR, Cross PL, Whitworth JA, et al (2006) Herpes simplex virus 2 infection increases HIV acquisition in men and women: systematic review and meta-analysis of longitudinal studies. AIDS 20: 73–83. 16327322 10.1097/01.aids.0000198081.09337.a7

[7] Celum C, Wald A, Lingappa JR, Magaret AS, Wang RS, et al (2010) Acyclovir and transmission of HIV-1 from persons infected with HIV-1 and HSV-2. The New England journal of medicine 362: 427–439. 20089951 10.1056/NEJMoa0904849 PMC2838503

[8] Celum C, Wald A, Hughes J, Sanchez J, Reid S, et al (2008) Effect of aciclovir on HIV-1 acquisition in herpes simplex virus 2 seropositive women and men who have sex with men: a randomised, double-blind, placebo-controlled trial. Lancet 371: 2109–2119. 18572080 10.1016/S0140-6736(08)60920-4 PMC2650104

[9] Abdool Karim Q, Abdool Karim SS, Frohlich JA, Grobler AC, Baxter C, et al (2010) Effectiveness and safety of tenofovir gel, an antiretroviral microbicide, for the prevention of HIV infection in women. Science 329: 1168–1174. 20643915 10.1126/science.1193748 PMC3001187

[10] Boily MC, Dimitrov D, Masse B (2011) How much of the overall microbicide effectiveness against HIV is due to the protection of TFV gel against HSV-2? The CAPRISA-004 trial. 18th Conference on Retroviruses and Opportunistic Infections. Boston, MA.

[11] Celum C, Morrow R, Donnell D, Hong T, Fife K, et al.. (2013) Daily oral tenofovir and emtricitabine/tenofovir pre-exposure prophylaxis and prevention of herpes simplex virus type 2 acquisition among heterosexual men and women. 20th Conference on Retroviruses and Opportunistic Infections. Atlanta, GA.

[12] Andrei G, Lisco A, Vanpouille C, Introini A, Balestra E, et al (2011) Topical tenofovir, a microbicide effective against HIV, inhibits herpes simplex virus-2 replication. Cell host & microbe 10: 379–389. 22018238 10.1016/j.chom.2011.08.015 PMC3201796

[13] Grant RM, Lama JR, Anderson PL, McMahan V, Liu AY, et al (2010) Preexposure chemoprophylaxis for HIV prevention in men who have sex with men. N Engl J Med 363: 2587–2599. 21091279 10.1056/NEJMoa1011205 PMC3079639

[14] LeGoff J, Mayaud P, Gresenguet G, Weiss HA, Nzambi K, et al (2008) Performance of HerpeSelect and Kalon assays in detection of antibodies to herpes simplex virus type 2. Journal of clinical microbiology 46: 1914–1918. 18385443 10.1128/JCM.02332-07 PMC2446869

[15] Anderson PL, Glidden DV, Liu A, Buchbinder S, Lama JR, et al (2012) Emtricitabine-tenofovir concentrations and pre-exposure prophylaxis efficacy in men who have sex with men. Sci Transl Med 4: 151ra125. 10.1126/scitranslmed.3004006 PMC3721979 22972843

[16] Rubin DB (1987) Multiple Imputation for Nonresponse in Surveys. New York: Wiley and Sons.

[17] White IR, Royston P, Wood AM (2011) Multiple imputation using chained equations: Issues and guidance for practice. Statistics in medicine 30: 377–399. 21225900 10.1002/sim.4067

[18] van de Laar MJ, Termorshuizen F, Slomka MJ, van Doornum GJ, Ossewaarde JM, et al (1998) Prevalence and correlates of herpes simplex virus type 2 infection: evaluation of behavioural risk factors. International journal of epidemiology 27: 127–134. 9563706 10.1093/ije/27.1.127

[19] Lupi O (2011) Prevalence and risk factors for herpes simplex infection among patients at high risk for HIV infection in Brazil. International journal of dermatology 50: 709–713. 21595666 10.1111/j.1365-4632.2010.04863.x

[20] Okuku HS, Sanders EJ, Nyiro J, Ngetsa C, Ohuma E, et al (2011) Factors associated with herpes simplex virus type 2 incidence in a cohort of human immunodeficiency virus type 1-seronegative Kenyan men and women reporting high-risk sexual behavior. Sexually transmitted diseases 38: 837–844. 21844740 10.1097/OLQ.0b013e31821a6225 PMC3157056

[21] Hendrix CW (2012) The clinical pharmacology of antiretrovirals for HIV prevention. Current opinion in HIV and AIDS 7: 498–504. 22964888 10.1097/COH.0b013e32835847ae PMC6986781

[22] Patterson KB, Prince HA, Kraft E, Jenkins AJ, Shaheen NJ, et al (2011) Penetration of tenofovir and emtricitabine in mucosal tissues: implications for prevention of HIV-1 transmission. Science translational medicine 3: 112re114. 10.1126/scitranslmed.3003174 PMC3483088 22158861

[23] Tan D (2012) Potential role of tenofovir vaginal gel for reduction of risk of herpes simplex virus in females. International journal of women's health 4: 341–350. 10.2147/IJWH.S27601 PMC3422111 22927765

[24] Hendrix CW, Chen BA, Guddera V, Hoesley C, Justman J, et al (2013) MTN-001: randomized pharmacokinetic cross-over study comparing tenofovir vaginal gel and oral tablets in vaginal tissue and other compartments. PLoS One 8: e55013. 23383037 10.1371/journal.pone.0055013 PMC3559346

[25] Tan DH, Kaul R, Raboud JM, Walmsley SL (2011) No impact of oral tenofovir disoproxil fumarate on herpes simplex virus shedding in HIV-infected adults. AIDS 25: 207–210. 21150556 10.1097/QAD.0b013e328341ddf7

[26] Golden MR, Ashley-Morrow R, Swenson P, Hogrefe WR, Handsfield HH, et al (2005) Herpes simplex virus type 2 (HSV-2) Western blot confirmatory testing among men testing positive for HSV-2 using the focus enzyme-linked immunosorbent assay in a sexually transmitted disease clinic. Sexually transmitted diseases 32: 771–777. 16314775 10.1097/01.olq.0000175377.88358.f3

